# Effect of vitamin D supplementation on clinical outcomes of patients with Parkinson disease: A randomized controlled study

**DOI:** 10.1097/MD.0000000000049931

**Published:** 2026-07-24

**Authors:** Chumpol Anamnart, Ram Kitjarak

**Affiliations:** a Division of Neurology, Department of Medicine, Prapokklao Hospital, Chanthaburi, Thailand.

**Keywords:** 25-hydroxyvitamin D level, Parkinson disease, vitamin D, vitamin D deficiency

## Abstract

**Background:**

Vitamin D deficiency is linked to Parkinson disease (PD) pathogenesis; however, clinical trial results remain inconclusive. Thus, in this study, we aimed to evaluate the prevalence of vitamin D deficiency in Thai patients with PD and the efficacy of vitamin D3 supplementation on clinical outcomes in a tropical, sun-abundant region.

**Methods:**

In this randomized controlled trial, we enrolled 60 patients with PD and 60 healthy controls. Patients with PD were randomized 1:1 to receive 2000 IU/d of vitamin D3 or no additional treatment (control) for 12 weeks. Primary outcomes included changes in MDS-UPDRS, Hoehn and Yahr (mH&Y), Thai Mini-Mental State Examination, and PDQ-39 scores. The secondary outcome was the prevalence of vitamin D deficiency (25(OH)D < 20 ng/mL).

**Results:**

At baseline, vitamin D deficiency was significantly more prevalent in patients with PD than in healthy controls (23.2% vs 10.0%, *P* = .046). After 12 weeks, the vitamin D group showed a significant increase in serum 25(OH)D levels compared to the control group (*P* < .001, successfully normalizing levels in the deficiency subgroup (30.96 ± 3.18 ng/mL). Although Thai Mini-Mental State Examination scores improved significantly within the vitamin D group (mean difference 1.37, *P* < .001), a similar improvement occurred in the control group (mean difference 0.93, *P* = .010), with no significant difference between groups (*P* = .378). No significant improvements were observed in motor function (MDS-UPDRS, mH&Y) or quality of life (PDQ-39).

**Conclusion:**

Vitamin D deficiency is common among Thai patients with PD, despite equatorial proximity. While 2000 IU/d of vitamin D3 safely corrected nutritional deficiency, it provided no significant short-term motor or cognitive benefits.

## 1. Introduction

Vitamin D is a lipophilic steroid hormone, and its primary source is vitamin D3 (cholecalciferol), which is produced in the skin in response to sun exposure. Dietary intake can provide small amounts of both vitamin D2 (ergocalciferol) and D3, which are metabolized in the liver and kidneys, respectively.^[1]^ A metabolized form of vitamin D in the liver, 25-hydroxyvitamin D (25(OH)D), is a biomarker of vitamin D status. Vitamin D plays a role in various cellular mechanisms via the vitamin D receptor (VDR), which is widely distributed throughout the skeletal and extraskeletal systems. Vitamin D deficiency is associated with osteomalacia and osteoporosis^[2]^ and can affect susceptibility to autoimmune and infectious diseases, cardiovascular disease, diabetes, and certain types of cancer.^[3-7]^ Additionally, some studies suggest that vitamin D deficiency may be a risk factor for the onset and progression of Parkinson disease (PD)^[8,9]^; however, the evidence is conflicting.^[10,11]^ A study conducted in Thailand, which we considered to be the most extensive study to date on vitamin D status, indicated that 45.2% of the population exhibited vitamin D insufficiency, that is, a serum 25(OH)D level below 30 ng/mL, while 5.7% of the population was classified as vitamin D-deficient, with serum 25(OH)D levels below 20 ng/mL.^[12]^ More recent data from a 2019–2020 national health examination survey reported that serum 25(OH)D levels were below 75 nmol/L in 31% of the population and below 50 nmol/L in 4%.^[13]^ These findings highlight a significant public health concern regarding vitamin D status in Thailand, despite its equatorial proximity.

Parkinson disease is a progressive neurodegenerative condition associated with the loss of dopaminergic neurons in the substantia nigra.^[14]^ Multiple pathways leading to neuronal death have been proposed; however, the exact mechanisms remain unknown. Vitamin D plays a role in dopamine synthesis by promoting the development of thyroxine hydroxylase-positive neurons.^[15]^ It also acts in dopamine release by increasing the production of synaptic vesicle glycoprotein 2C, a presynaptic release protein.^[15]^ In addition, vitamin D affects the synthesis of neurotrophic factors, such as glial cell line-derived neurotrophic factor and brain-derived neurotrophic factor, necessary for neurogenesis and dopaminergic neuron survival.^[16-18]^ Moreover, evidence suggests that vitamin D attenuates oxidative stress by modulating reactive oxygen species production.^[19]^ Oxidative stress may drive the loss of dopaminergic neurons.^[20,21]^ Notably, VDR and 1-α-hydroxylase, responsible for converting vitamin D from the inactive into active form, are highly expressed in dopaminergic neurons of the substantia nigra.^[22]^ A postmortem study of human brains demonstrated that the 1-α-hydroxylase level was significantly reduced in dopaminergic neurons of the substantia nigra. In contrast, no difference was observed in VDR expression between patients with PD and healthy controls.^[23]^ These findings suggest that active vitamin D levels are reduced in dopaminergic neurons of patients with PD.

Several studies have shown that low vitamin D levels increase the risk of PD and are associated with the severity of both motor and non-motor symptoms.^[24-31]^ However, only a few studies have examined the effect of vitamin D supplementation on the improvement in clinical symptoms in patients with PD, and the results remain inconclusive. Thus, in this study, we aimed to evaluate the effect of vitamin D3 supplementation on the clinical outcomes for patients with PD.

## 2. Materials and methods

This was a randomized, parallel, controlled trial conducted at King Prajadhipok Memorial Hospital (Prapokklao Hospital), Thailand, between January and October 2024. The study was registered with the Thai Clinical Trials Registry (TCTR20240708007). URL: https://www.thaiclinicaltrials.org/show/TCTR20240708007. While the registration was performed retrospectively, the authors confirm that the trial protocol remained unchanged from the start of data collection, and all procedures were approved by the Chanthaburi Research Ethics Committee (Reference Number: CTREC 001/67) prior to participant enrollment. All methods adhered to relevant guidelines and regulations as well as to the Consolidated Standards of Reporting Trials (CONSORT) guidelines.

### 2.1. Study population and inclusion and exclusion criteria

The study population consisted of patients with PD aged ≥50 years who met the clinical diagnostic criteria of the UK Brain Bank for PD. The healthy control population included healthy individuals aged ≥50 years. The following individuals were excluded from the study: patients currently using vitamin D, patients diagnosed with stones in the urinary tract, patients diagnosed with osteoporosis or bone fractures, patients with moderate to severe dementia (defined by a Thai version of the Mini-Mental State Examination [TMMSE] score ≤15), and patients with severe psychosis. Patients provided written informed consent before data collection. The sample size calculation was based on the Unified Parkinson Disease Rating Scale (UPDRS) primary outcome. A total of 60 participants were required to achieve 80% power at a significance level of 0.05 (see Supplemental Digital Content 1, Supplemental Digital Content 1, which details the sample size determination).

### 2.2. Randomization and intervention

We used computer-generated blocked randomization to allocate 60 patients with PD into the following 2 groups (30 patients per group): vitamin D group, wherein patients received 2000 IU of vitamin D3 once daily, and control group, wherein patients received no additional medications. Therapy was continued for 12 weeks, and no additional dopaminergic or non-dopaminergic treatment was allowed during this period.

To assess clinical outcomes, assessments were performed using the UPDRS,^[32]^ modified Hoehn and Yahr scale (mH&Y),^[33]^ TMMSE validated by Puangvarin & Train the Brain Forum,^[34]^ and Parkinson Disease Questionnaire (PDQ-39)^[35]^ by neurologists at baseline and week 12. The serum 25(OH)D level was measured at baseline and week 12 using the chemiluminescent microparticle immunoassay method (Abbott, Architect i100SR). Moreover, we compared the serum 25(OH)D levels of the study population (60 patients with PD) with those of 60 healthy controls. Low 25(OH)D levels were categorized into: vitamin D insufficiency, defined by a 25(OH)D level 20 to 30 ng/mL, and vitamin D deficiency, defined by a 25(OH)D level <20 ng/mL. Vitamin D sufficiency was defined as a 25(OH)D level >30 ng/mL.

The primary outcomes were clinical differences from baseline after vitamin D3 supplementation, assessed using the UPDRS, mH&Y, TMMSE, and PDQ-39. The secondary outcome was the prevalence of vitamin D deficiency in patients with PD.

### 2.3. Statistical analyses

Data on the general and clinical characteristics of the sample were analyzed using descriptive statistics. Qualitative variables were reported as frequencies and percentages and compared using the chi-square or Fisher exact test. Quantitative variables were reported as means and standard deviations and compared using the independent-samples *t* test or Mann–Whitney *U* test, as suitable.

Mean differences in the UPDRS, mH&Y, TMMSE, and PDQ-39 scores and 25(OH)D levels between pre- and post-intervention (within groups) timepoints were compared using paired *t* tests. Differences in mean 25(OH)D levels between patients with PD and healthy controls were compared using independent-samples *t* tests, and mean differences between groups were estimated using a generalized linear model. The proportion of vitamin D deficiency was compared between patients with PD and healthy controls using a generalized linear model with a Gaussian family and 95% confidence interval. Data were analyzed using SPSS version 29.0 software (IBM Corp, Armonk). Statistical significance was set at *P*-values <.05.

## 3. Results

**Figure 1.**
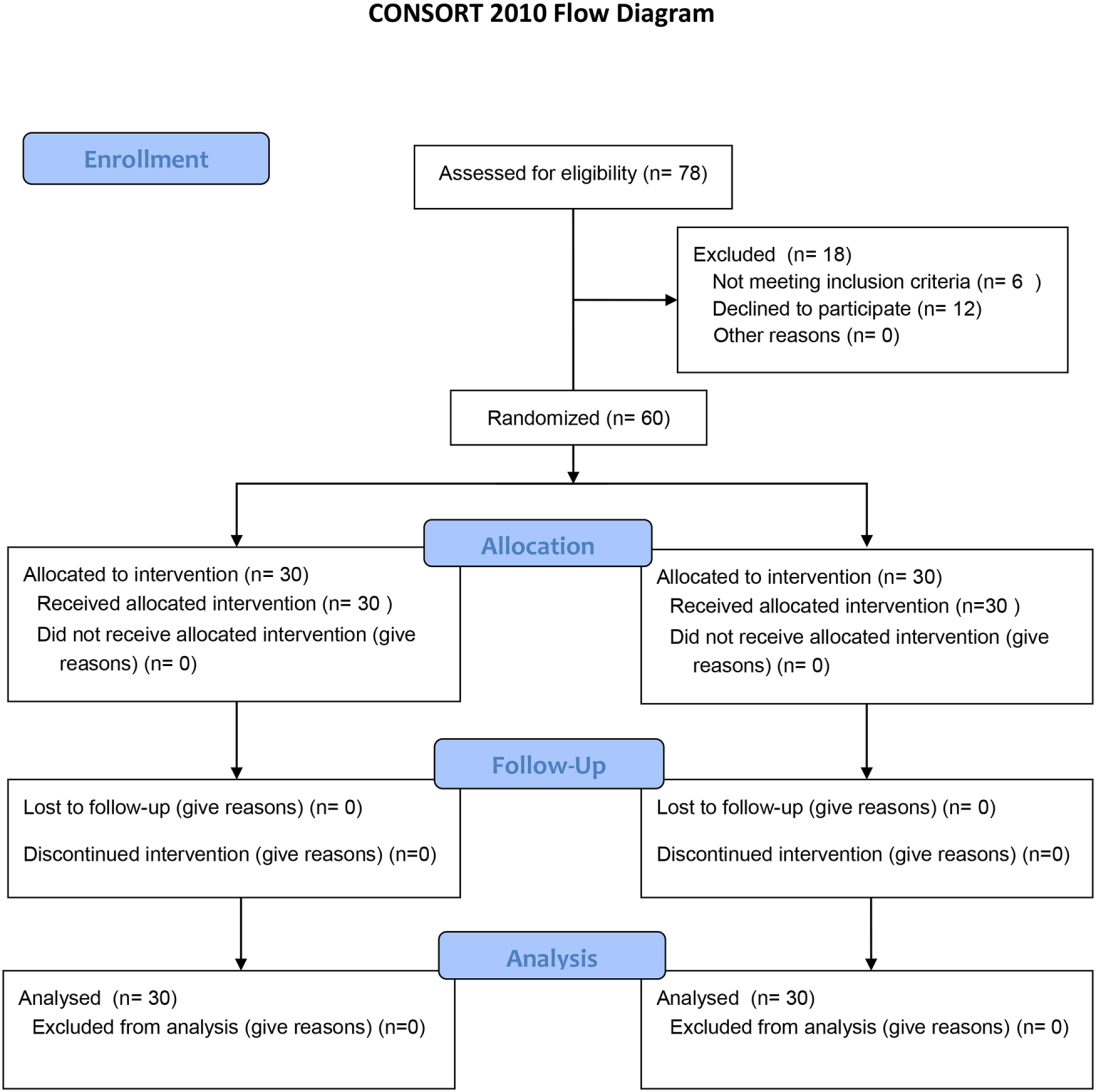
Study participants’ flow diagram. Illustrated are the exclusion and inclusion criteria and the study flow chart.

A total of 78 patients were assessed for eligibility, of whom 60 were enrolled; 30 received vitamin D3 and 30 did not. No patients dropped out of the study; therefore, all participants were included in the analysis. No patient reported any side effects of vitamin D3. The participant flow diagram is presented in Figure 1. The baseline characteristics of participants are shown in Table 1.

**Table 1:** Baseline characteristics of study participants.

Characteristic	Vitamin D (n = 30)	Control (n = 30)	*P*-value
Age (yr)	69.33 ± 10.26	68.80 ± 9.30	.834*
Sex			
Female	17 (56.7)	13 (43.3)	.302‡
Male	13 (43.3)	17 (56.7)	
Comorbidity			
Diabetes mellitus	3 (10.0)	4 (13.3)	1.000§
Hypertension	12 (40.0)	18 (60.0)	.121‡
Dyslipidemia	8 (26.7)	15 (50.0)	.063‡
Stroke	1 (3.3)	1 (3.3)	1.000§
Disease duration (mo)	68 (36–96)	48 (24–84)	.161†
Levodopa dose (mg/d)	300 (300–600)	300 (150–450)	.459†
Modified H&Y	2.25 ± 0.92	2.12 ± 0.87	.565*
UPDRS			
Total score	40.33 ± 24.11	31.73 ± 22.64	.160*
Part I	5.80 ± 3.23	4.57 ± 2.91	.126*
Part II	11.57 ± 7.46	9.53 ± 6.69	.271*
Part III	22.63 ± 15.58	16.70 ± 14.73	.135*
Part IV	0.43 ± 1.65	0.93 ± 2.96	.422*
TMMSE	25.83 ± 3.35	25.83 ± 3.29	1.000*
PDQ-39	19.20 ± 14.25	16.91 ± 16.03	.561*
25(OH)D level, (ng/mL)	25.03 ± 9.55	27.72 ± 9.34	.274*
Sufficiency	9 (30.0)	11 (36.7)	.437‡
Insufficiency	12 (40.0)	14 (46.7)	
Deficiency	9 (30.0)	5 (16.7)	
Vitamin D deficiency	9 (30.0)	5 (16.7)	.222‡

Data are presented as count (%), mean ± standard deviation or median (interquartile range).

Modified H&Y = Modified Hoehn and Yahr scale, PDQ-39 = Parkinson Disease Questionnaire, TMMSE = Thai version of the Mini-Mental State Examination Score, UPDRS = Unified Parkinson Disease Rating Scale.

* *P*-value corresponds to that from independent-samples *t* test.

† *P*-value corresponds to that from Mann–Whitney *U* test.

‡ *P*-value corresponds to that from chi-square test.

§ *P*-value corresponds to that from Fisher’s exact test.

No significant differences were noted in age, sex, comorbidities, disease duration, levodopa dose, the mH&Y, UPDRS, TMMSE, and PDQ-39 scores, and 25(OH)D levels between the vitamin D and control groups. The effects of vitamin D supplementation on clinical outcomes between the vitamin D and control groups are presented in Table 2. No significant differences were observed in the mH&Y, UPDRS, TMMSE, and PDQ-39 scores between the 2 groups. However, the 25(OH)D levels were significantly higher in the vitamin D group than in the control group.

**Table 2:** Comparison of outcomes and 25(OH)D levels between the vitamin D and control groups.

Outcome	Vitamin D (n = 30), mean ± SD	Control (n = 30), mean ± SD	Mean difference (95% CI)	*P*-value*
Modified H&Y UPDRS	2.28 ± 0.68	2.07 ± 0.83	0.22 (−0.17, 0.61)	.272
Total score	37.40 ± 23.08	29.43 ± 20.76	7.97 (−3.38, 19.31)	.165
Part I	5.23 ± 3.55	5.07 ± 2.85	0.17 (−1.50, 1.83)	.842
Part II	12.57 ± 8.68	9.90 ± 7.32	2.67 (−1.48, 6.82)	.203
Part III	19.53 ± 14.08	14.63 ± 12.99	4.90 (−2.10, 11.90)	.167
Part IV	0.40 ± 1.55	0.00 ± 0.00	0.40 (−0.18, 0.98)	.167
TMMSE	27.20 ± 2.48	26.77 ± 2.90	0.43 (−0.96, 1.83)	.536
PDQ-39	17.53 ± 19.38	14.71 ± 14.94	2.82 (−6.12, 11.76)	.530
25(OH)D level, (ng/mL)	37.55 ± 10.58	26.21 ± 8.80	11.34 (6.32, 16.37)	<.001

CI = confidence interval, Modified H&Y = Modified Hoehn and Yahr scale, PDQ-39 = Parkinson Disease Questionnaire, TMMSE = Thai version of the Mini-Mental State Examination Score, UPDRS = Unified Parkinson Disease Rating Scale.

* *P*-value corresponds to that from the independent-samples *t* test.

The effects of vitamin D on clinical outcomes were compared within each group (Table 3). No significant differences were observed in mH&Y, UPDRS, and PDQ-39 scores within each group. The TMMSE scores significantly improved within each group (mean difference within groups: 1.37 vs 0.93; *P* < .001 vs *P* = .010 in the vitamin D and control groups, respectively). However, the mean difference in TMMSE scores did not reach statistical significance between groups (mean difference = 0.43, *P* = .378; Table 4).

**Table 3:** Comparison of outcomes and 25(OH)D levels within groups.

Outcome	0 wk, mean ± SD	12 wk, mean ± SD	Mean difference (95% CI)		*P*-value
Modified H&Y					
Vitamin D group	2.25 ± 0.92	2.28 ± 0.68	0.03	(−0.16, 0.23)	.730
Control group	2.12 ± 0.87	2.07 ± 0.83	−0.05	(−0.22, 0.12)	.557
UPDRS					
Total score					
Vitamin D group	40.33 ± 24.11	37.40 ± 23.08	−2.93	(−6.88, 1.01)	.139
Control group	31.73 ± 22.64	29.43 ± 20.76	−2.30	(−6.29, 1.69)	.248
Part I					
Vitamin D group	5.80 ± 3.23	5.23 ± 3.55	−0.57	(−1.71, 0.58)	.319
Control group	4.57 ± 2.91	5.07 ± 2.85	0.50	(−0.65, 1.65)	.382
Part II					
Vitamin D group	11.57 ± 7.46	12.57 ± 8.68	1.00	(−0.55, 2.55)	.198
Control group	9.53 ± 6.69	9.90 ± 7.32	0.37	(−1.37, 2.11)	.669
Part III					
Vitamin D group	22.63 ± 15.58	19.53 ± 14.08	−3.10	(−6.68, 0.48)	.087
Control group	16.70 ± 14.73	14.63 ± 12.99	−2.07	(−4.71, 0.58)	.121
Part IV					
Vitamin D group	0.43 ± 1.65	0.40 ± 1.55	−0.03	(−0.58, 0.51)	.901
Control group	0.93 ± 2.96	0.00 ± 0.00	−0.93	(−2.04, 0.17)	.095
TMMSE					
Vitamin D group	25.83 ± 3.35	27.20 ± 2.48	1.37	(0.65, 2.08)	.001
Control group	25.83 ± 3.29	26.77 ± 2.90	0.93	(0.24, 1.63)	.010
PDQ-39					
Vitamin D group	19.20 ± 14.25	17.53 ± 19.38	−1.68	(−7.91, 4.55)	.586
Control group	16.91 ± 16.03	14.71 ± 14.94	−2.21	(−6.97, 2.56)	.351
25(OH)D level, (ng/mL)					
Vitamin D group	25.03 ± 9.55	37.55 ± 10.58	12.53	(9.77, 15.28)	<.001
Control group	27.72 ± 9.34	26.21 ± 8.80	−1.51	(−3.31, 0.30)	.099

CI = confidence interval, Modified H&Y = Modified Hoehn and Yahr scale, PDQ-39 = Parkinson Disease Questionnaire, SD = standard deviation, TMMSE = Thai version of the Mini-Mental State Examination Score, UPDRS = Unified Parkinson Disease Rating Scale.

**Table 4:** Comparison of the mean difference of outcomes and 25(OH)D levels between the vitamin D and control groups.

Outcome	Vitamin D (n = 30), mean ± SD	Control (n = 30), mean ± SD	Mean difference (95% CI)	*P*-value*
Modified H&Y	0.03 ± 0.52	−0.05 ± 0.46	0.08 (−0.17, 0.34)	.516
UPDRS				
Total score	−2.93 ± 10.56	−2.3 ± 10.68	−0.63 (−6.12, 4.86)	.818
Part I	−0.57 ± 3.06	0.50 ± 3.08	−1.07 (−2.65, 0.52)	.184
Part II	1.00 ± 4.16	0.37 ± 4.66	0.63 (−1.65, 2.92)	.581
Part III	−3.10 ± 9.60	−2.07 ± 7.09	−1.03 (−5.39, 3.33)	.637
Part IV	−0.03 ± 1.45	−0.93 ± 2.96	0.9 (−0.31, 2.11)	.142
TMMSE	1.37 ± 1.92	0.93 ± 1.86	0.43 (−0.54, 1.41)	.378
PDQ-39	−1.68 ± 16.68	−2.21 ± 12.76	0.53 (−7.15, 8.21)	.891
Vitamin D (25(OH)D) level, (ng/mL)	12.53 ± 7.38	−1.51 ± 4.84	14.03 (10.8,17.27)	<.001

CI = confidence interval, Modified H&Y = Modified Hoehn and Yahr scale, PDQ-39 = Parkinson Disease Questionnaire, SD = standard deviation, TMMSE = Thai version of the Mini-Mental State Examination.

* *P*-value corresponds to that from the independent-samples *t* test.

**Figure 2.**
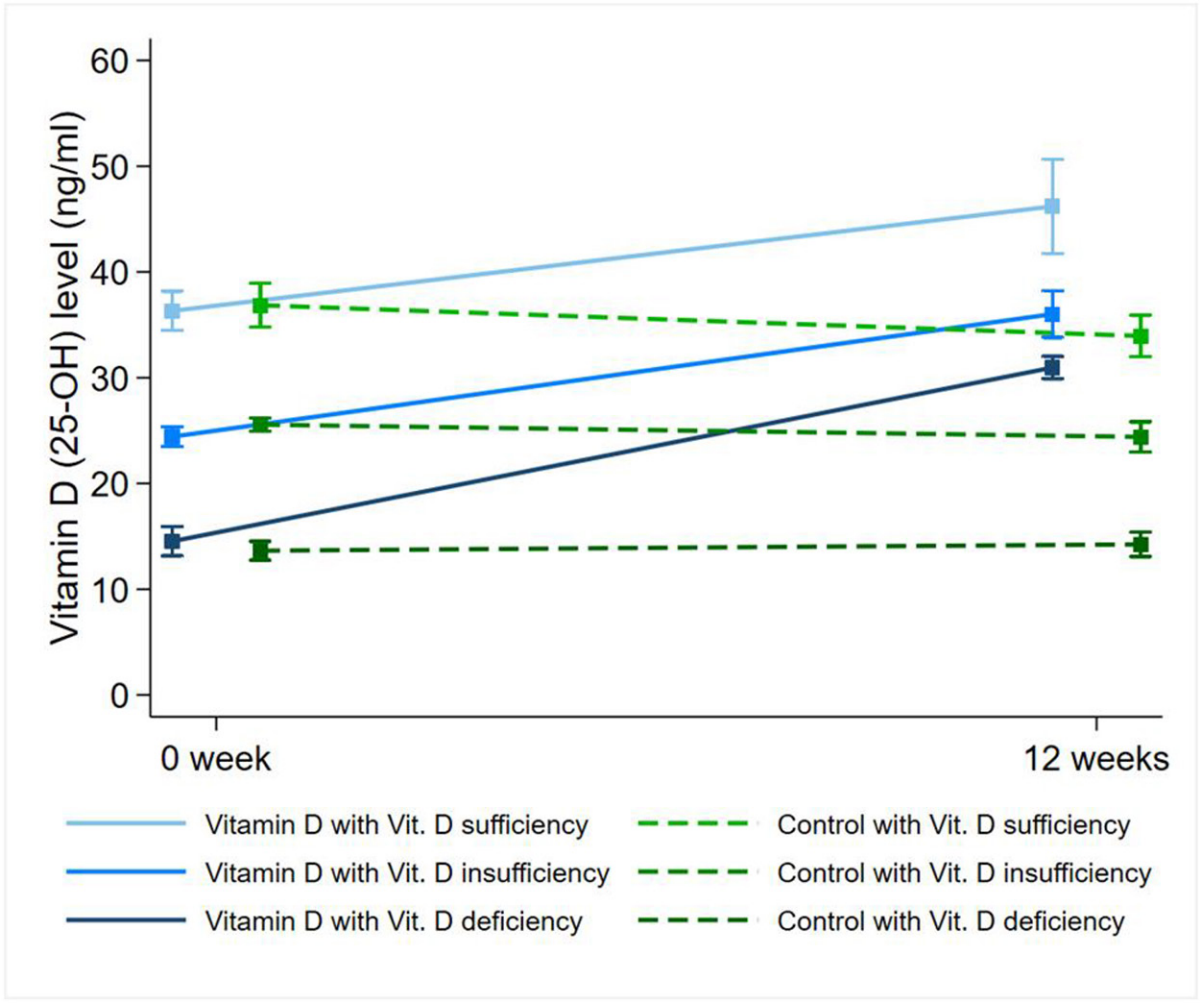
25(OH)D levels at baseline and week 12, stratified by baseline. Data show that the 25(OH)D levels significantly increased at 12 weeks following vitamin D3 supplementation in all vitamin D status subgroups.

The 25(OH)D levels within each group according to vitamin D status at baseline, sufficiency, insufficiency, and deficiency are presented in Table 5. Within the vitamin D group, the 25(OH)D levels at 12 weeks after intervention were significantly increased in the vitamin D status subgroups (Fig. 2). The most significant increase in 25(OH)D levels was observed in participants with vitamin D deficiency who received vitamin D3; the average (mean ± standard deviation) pre- and posttreatment 25(OH)D levels were 14.53 ± 4.13 and 30.96 ± 3.18, respectively (*P* < .001).

**Table 5:** Comparison of 25(OH)D levels within groups according to vitamin D status subgroups at baseline.

25(OH)D levels, (ng/ mL)	0 wk, mean ± SD	12 wk, mean ± SD	Mean difference (95% CI)	*P*-value*
Sufficiency (n = 20)				
Vitamin D group	36.32 ± 5.57	46.20 ± 13.36	9.88 (2.96, 16.80)	.011
Control group	36.86 ± 6.85	33.95 ± 6.52	−2.91 (−7.23,1.41)	.164
Insufficiency (n = 26)				
Vitamin D group	24.43 ± 3.23	36.02 ± 7.64	11.59 (7.25, 15.93)	<.001
Control group	25.56 ± 2.29	24.41 ± 5.37	−1.16 (−3.43, 1.11)	.291
Deficiency (n = 14)				
Vitamin D group	14.53 ± 4.13	30.96 ± 3.18	16.42 (12.5, 20.35)	<.001
Control group	13.64 ± 1.98	14.24 ± 2.58	0.60 (−2.03, 3.23)	.560

CI = confidence interval, SD = standard deviation.

* *P*-value corresponds to that from the paired-samples *t* test.

No significant difference in sex was observed between patients with PD and healthy controls (male: female = 1:1 in each group). However, patients in the PD group were significantly older than those in the healthy control group (mean ± SD: 69.07 ± 9.71 vs 63.67 ± 8.24 years; *P* = .001; Table 6). Vitamin D deficiency was significantly more common in the PD group than in the healthy control group (14 [23.3%] vs 6 [10.0%], respectively; *P* = .046; Table 7).

**Table 6:** Characteristics of patients with Parkinson disease and healthy controls.

Variable	Patients with Parkinson disease (n = 60)	Healthy controls (n = 60)	*P*-value
Age (yr)	69.07 ± 9.71	63.67 ± 8.24	.001*
Sex			
Female	30 (50.0)	30 (50.0)	1.000†
Male	30 (50.0)	30 (50.0)	

Data are presented as number (%), mean ± standard deviation.

* *P*-value corresponds to that from the independent-samples *t* test.

† *P*-value corresponds to that from the chi-square test.

**Table 7:** Comparison of 25(OH)D levels between patients with Parkinson disease and healthy.

Variable	Patients with Parkinson disease (n = 60)	Healthy controls (n = 60)	Absolute difference (95% CI)	*P*-value
25(OH)D level, (ng/mL)	26.37 ± 9.46	27.58 ± 7.76	−1.20 (−4.30, 1.89)*	.446
Vitamin D deficiency	14 (23.3)	6 (10.0)	13.33 (0.21, 26.45)†	.046

Data are presented as number (%), mean ± standard deviation.

* Absolute difference is the mean difference estimated using a generalized linear model with a Gaussian family.

† Absolute difference is the difference in proportions from a risk difference regression model (generalized linear model with an identity link in the binomial family).

## 4. Discussion

Our study reinforces the association between vitamin D status and PD within the Thai population. Patients with PD had a substantially higher prevalence of vitamin D deficiency (23.3%) than healthy controls (10.0%), even in a tropical, sun-abundant region. It is worth noting that while the mean serum 25(OH)D levels appeared comparable between the PD and control groups, the categorical analysis revealed a significantly higher proportion of vitamin D deficiency (<20 ng/mL) among patients with PD. This statistical divergence suggests that although the mathematical averages of the 2 cohorts were close, individuals with PD are more susceptible to clustering at the extreme lower end of vitamin D status. Therefore, evaluating clinical datasets using both continuous means and categorical thresholds provides a more comprehensive understanding of nutritional deficiencies in this population. This finding emphasizes that equatorial proximity does not inherently protect against hypovitaminosis D in neurodegenerative populations.

Several factors may explain this lack of clinical improvement. First, our 12-week study duration might have been insufficient to detect disease-modifying effects or meaningful clinical recovery. Neuroprotective mechanisms, such as the modulation of neurotrophic factors (GDNF/BDNF) and attenuation of oxidative stress in the substantia nigra, likely require sustained vitamin D sufficiency to translate into observable clinical improvement.^[36]^ Second, while 25(OH)D levels in the deficiency subgroup increased substantially, the levels remained within the “low-normal” range (approximately 30.96 ng/mL). A higher therapeutic threshold of serum vitamin D may be required to trigger meaningful dopaminergic neuroprotection.

Regarding cognitive function, although the TMMSE scores improved significantly within the vitamin D group, a similar improvement was observed in the control group. This finding suggests that the change may be attributed to a practice effect (i.e., improvement due to familiarity with the test) or the increased clinical attention to participants during the trial rather than a direct pharmacological effect of vitamin D.^[37]^

The primary strength of this study lies in its randomized controlled design, providing robust evidence on the efficacy of vitamin D3 supplementation in a regional cohort. We used standardized, internationally recognized tools (MDS-UPDRS, PDQ-39) to ensure the comparability with global data. Furthermore, our findings highlight the marked prevalence of vitamin D deficiency in a tropical climate, challenging the assumption that sun exposure alone is sufficient for patients with PD.

Nonetheless, this study has some limitations. As discussed earlier, the 12-week intervention period may be too short to capture slow, progressive changes in neurodegeneration or long-term clinical stabilization. Additionally, VDR gene polymorphisms, which are known to mediate the biological response to vitamin D and could explain individual variations in clinical outcomes,^[38]^ were not analyzed. Finally, adherence was based on patient self-reports; dietary vitamin D intake or incidental sunlight exposure were not strictly controlled, potentially confounding the observed serum vitamin D levels.

In conclusion, our study confirms that patients with PD in Thailand have a significantly higher prevalence of vitamin D deficiency than healthy individuals. While daily administration of 2000 IU of vitamin D3 for 12 weeks effectively normalized serum 25(OH)D levels, particularly in those with baseline deficiency, it did not significantly improve motor function, cognitive scores, or quality of life within this timeframe. These findings suggest that, while vitamin D supplementation is a safe and effective strategy to correct nutritional deficiencies in patients with PD, its role as a symptomatic or disease-modifying treatment in the short term remains unestablished. Clinicians should monitor vitamin D levels as part of routine PD care to maintain bone health. However, further large-scale, long-term trials including genetic analysis are required to determine whether a “therapeutic window” exists in which vitamin D can influence PD progression.

## Author contributions

**Conceptualization:** Chumpol Anamnart.

**Data curation:** Chumpol Anamnart, Ram Kitjarak.

**Formal analysis:** Chumpol Anamnart.

**Investigation:** Chumpol Anamnart, Ram Kitjarak.

**Methodology:** Chumpol Anamnart.

**Writing – original draft:** Chumpol Anamnart.

**Writing – review & editing:** Chumpol Anamnart, Ram Kitjarak.


