# Supplementary material for: Effect of vitamin D supplementation on clinical outcomes of patients with Parkinson disease: A randomized controlled study
Source: Medicine (Baltimore). 2026 Jul 24;105(30):e49931. doi: 10.1097/MD.0000000000049931 (PMC13406316; doi:10.1097/MD.0000000000049931)
Supplement: Supplementary file 1 [file medi-105-e49931-s001.docx]

**Sample size determination**

This study aimed to evaluate the effect of vitamin D3 supplementation on the clinical outcomes of patients with Parkinson’s disease. By comparing the means of the Unified Parkinson's Disease Rating Scale (UPDRS), modified Hoehn and Yahr scale, Mini Mental State Examination, and Parkinson's Disease Questionnaire-39 between the experimental and control groups, we considered using the UPDRS as the primary outcome. The sample size was determined using the sample size calculation formula for comparing the means of two groups (testing two independent means) as follows:

$${n_{1}}=\frac{\left( Z_{1-\alpha/2}+Z_{1-\beta} \right)^{2}2\sigma^{2}}{\Delta^{2}}$$

- n1 is the number of samples of the experimental group.
- Z1-α/2 is the standard statistic under the normal curve corresponding to the significance level. The significance level is set at α = 0.05, so Z1-α = 1.96.
- Z1-β is the standard statistic under the normal curve corresponding to the test power. The test power is set at 80 percent, so Z1-β = 0.842.
- ∆ is the difference in means between the experimental and control groups.

∆=μ_1-μ_2

We set the difference in the total UPDRS score that changed between the experimental group and the control group (The minimal clinically significant difference, MCSD) equal to 10 points (10% change of UPDRS total score (0- 195))

- σ2 is population variance, estimated using the sample variance. It is based on a study of the standard deviation of the sample from a randomized, double-blind, placebo-controlled trial of vitamin D supplementation in Parkinson’s disease (1). The pooled standard deviation of the total UPDRS score change was 13.69 points. We therefore set the sample standard deviation to 13.0 when calculating the sample size.

The values ​​can be substituted into the sample size calculation formula as follows:

*n_1_ = (1.96 + 0.842)^2^ 2(13)^2^*

*(10)^2^*

*n_1_ = 27*

The calculation yielded a minimum requirement of 27 participants per group. To account for a potential 10% attrition or loss to follow-up rate, the sample size was adjusted using the formula: (n_new_ = 27 / (1 – 0.10)). This resulted in a required sample size of 30 participants per group. Therefore, 60 participants (30 in the experimental group and 30 in the control group) were enrolled in this study.

**Reference**

1. Suzuki M, Yoshioka M, Hashimoto M, Murakami M, Noya M, Takahashi D, et al. Randomized, double-blind, placebo-controlled trial of vitamin D supplementation in Parkinson disease. Am J Clin Nutr (2013) 97:1004–13. doi: [10.3945/ajcn.112.051664](https://doi.org/10.3945/ajcn.112.051664).
